# Qinwen Baidu decoction for sepsis: A protocol for a systematic review and meta-analysis

**DOI:** 10.1097/MD.0000000000014761

**Published:** 2019-03-01

**Authors:** Ping Wang, Hefei Huang, Jingya Zhong, Hairong Cai, Yonglian Huang, Dongjie Chen, Yaxiu Huang, Shaoping Li, Qifeng Cao, Xiaohong Peng

**Affiliations:** a Department of Critical Care Medicine, Beijing University of Chinese Medicine Shenzhen Hospital; b The Second Clinical Medical School, Guangzhou University of Chinese Medicine, Guangzhou, Guangdong Province, China.

**Keywords:** protocol, Qingwen Baidu decoction, sepsis, systematic review

## Abstract

**Background:**

Sepsis is the most common critical illness in the clinic, with a high incidence and mortality. Qingwen Baidu decoction (QWBDD) has been widely applied in the treatment of sepsis, however, there is no systematic review or meta-analysis of QWBDD in the treatment of sepsis. Hence, we provide a protocol of systematic review and meta-analysis to evaluate the efficacy and safety of QWBDD in the treatment of sepsis.

**Methods:**

The databases including Cochrane Library, PubMed, Embase, Web of Science, Cochrane Clinical Trial Database, World Health Organization International Clinical Trial Registration Platform, CNKI, CBM, VIP, and WanFang Database will be searched from the time when the respective databases were established to January 2019. All randomized controlled trials (RTCs) published in Chinese and English assessing QWBDD for sepsis will be included. Continuity data are expressed as mean difference (MD) or standard mean difference (SMD), and dichotomous data is expressed as relative risk. Analyses will be performed by using RevMan V.5.3.5 software.

**Results:**

This study will provide high-quality synthesis of current evidence of QWBDD in the treatment of sepsis from the following aspects, including 28-day mortality, mean arterial pressure (MAP), blood lactate, procalcitonin (PCT), tumor necrosis factor-α (TNF-α), interleukin-6 (IL-6), hypersensitive C-reactive protein (hs-CRP), acute physiology and chronic health score (APACHE-II), intensive care unit stay, mean hospital stay, mechanical ventilation time, etc.

**Conclusion:**

Our systematic review will provide evidence for judging whether QWBDD is an effective intervention for sepsis.

**PROSPERO registration number:**

PROSPERO CRD 42019123078.

## Abstract 2

Supplemental Digital Content is available in the text

## 1 Introduction

Sepsis is a life-threatening organ dysfunction caused by a dysregulated host response to infection and still the leading cause of death in critically ill patients.^[1]^ The incidence rate of sepsis is as high as 10% to 40% and rises at a rate of 1.5% per year. Severe sepsis accounts for 10% of intensive care unit (ICU) inpatients and results in a 20% to 52% mortality rate, and the mortality rate of septic shocks could even be as high as 40% to 70%,^[4–10]^ which is the leading cause of death secondary to coronary artery disease in European and American countries.^[2]^ With the aging of the population, the increase of drug-resistant pathogenic microorganisms, human medical activities, and immunosuppressed patients, the incidence of sepsis is on the rising.^[3]^ There are nearly 750,000 patients with severe sepsis and septic shock in the United States each year, and the annual medical cost of treating sepsis is as high as $16.7 billion.^[11]^ In China, the cost of treatment per day for every sepsis patient is as high as $502.^[12]^ Although the International Guidelines for Management of Sepsis and Septic Shock has been continuously updated and many new technologies for the treatment of sepsis have been emerged,^[1,13,14]^ no effective methods have been found to reduce the incidence and mortality of sepsis.^[15,16]^

Traditional Chinese medicine (TCM) is an important part of complementary and alternative medicine (CAM), which has been widely applied in clinical practice in China.^[17]^ Qingwen Baidu decoction (QWBDD) is comprised of 15 kinds of TCM (*Rehmannia glutinosa* Libosch, Coptidis Rhizoma, *Scutellaria baicalensis* Georgi, Cortex Moutan, Gypsum Fibrosum, *Gardenia jasminoides* Ellis, *Glycyrrhiza uralensis* Fisch, *Lophatherum gracile*, *Scrophularia ningpoensis* Hemsl, *Rhinoceros unicornis* L, *Forsythia suspensa*, *Paeonia lactiflora* Pall, *Anemarrhena asphodeloides* Bunge, *Platycodon grandiflorus*). QWBDD has been used widely in clinical practice to treat sepsis with a certain effect by some low quality randomized controlled trials (RCTs) published in China.^[18–21]^ However, there have been few systematic reviews regarding effectiveness and safety of QWBDD in the treatment of sepsis. Therefore, we provide a protocol of systematic review and meta-analysis to evaluate the effectiveness and safety of QWBDD for sepsis, in order to provide a stronger evidence-based medical basis for clinical application.

## 2 Methods

### 2.1 Inclusion criteria for study selection

#### 2.1.1 Types of studies

All the RCTs of QWBDD in the treatment of sepsis will be included, the language is limited to Chinese and English, regardless of whether blinding or allocation concealment was adopted. Non-randomized controlled trials, animal experiments, case or empirical reports, reviews, abstracts, and repeated publications will be excluded.

#### 2.1.2 Types of patients

Patients diagnosed as sepsis (older than 18 years) according to the Third International Consensus Definitions for Sepsis and Septic Shock (Sepsis-3) developed by Society of Critical Care Medicine and European Society of Intensive Care Medicine will be included.^[22]^ There is no limitation to patient's age, sex, ethnicity, and severity of the disease. Patients with HIV infection, malignant tumors, connective tissue disease or immune system diseases will be excluded.

#### 2.1.3 Types of interventions

The control group used the conventional treatment recommended in the guidelines^[1]^ (clearing the infection, early fluid resuscitation, anti-infective treatment, using vasoactive drugs, renal replacement therapy, using glucocorticoids, mechanical ventilation, etc.), and combined treatment of QWBDD and conventional treatment was used in the experimental group. The conventional treatment in the control group should be consistent with that of the experimental group. The dosage and route of administration of QWBDD are not limited. The course of treatment was at least 7 days and follow-up time was at least 28 days. However, there were no other TCM, acupuncture, acupoint sticking in both groups.

#### 2.1.4 Types of outcome measures

##### 2.1.4.1 Primary outcomes

The mortality rate of 28 days.

##### 2.1.4.2 Secondary outcomes

Secondary outcomes will include central venous pressure (CVP), mean arterial pressure (MAP), urine volume, superior vena cava oxygen saturation (ScvO2), mixed venous oxygen saturation (SvO2), lactate clearance (LAC), blood lactate, procalcitonin (PCT), tumor necrosis factor-α (TNF-α), interleukin-6 (IL-6), hypersensitive C-reactive protein (hs-CRP), acute physiology and chronic health score (APACHE-II), ICU stay, mean hospital stay, mechanical ventilation time, adverse reactions.

### 2.2 Search methods for the identification of studies

We will search the following databases: The Cochrane Library, PubMed, Embase, Web of Science, Cochrane Clinical Trial Database, World Health Organization International Clinical Trial Registration Platform (ClinicalTrials), China National Knowledge Infrastructure (CNKI), Chinese Biomedical Literature Database (CBM), Chinese Science and Technology Periodical Database (VIP), and WanFang Database from the time when the respective databases were established to January 2019. The search terms include:QWBDD, sepsis, and RCTs. The English databases will be searched according to the keywords of each database combined with free words. The strategy for searching the PubMed will be shown as an example in Appendix A (Supplemental Appendix A), and modified by using other databases.

#### 2.2.1 Searching other resources

We will search the references, related conference papers, or dissertations included in the study manually. In addition, relevant unpublished research results will be requested from other researchers or pharmaceutical manufacturers.

### 2.3 Data collection and analysis

#### 2.3.1 Selection of studies

**Figure 1:**
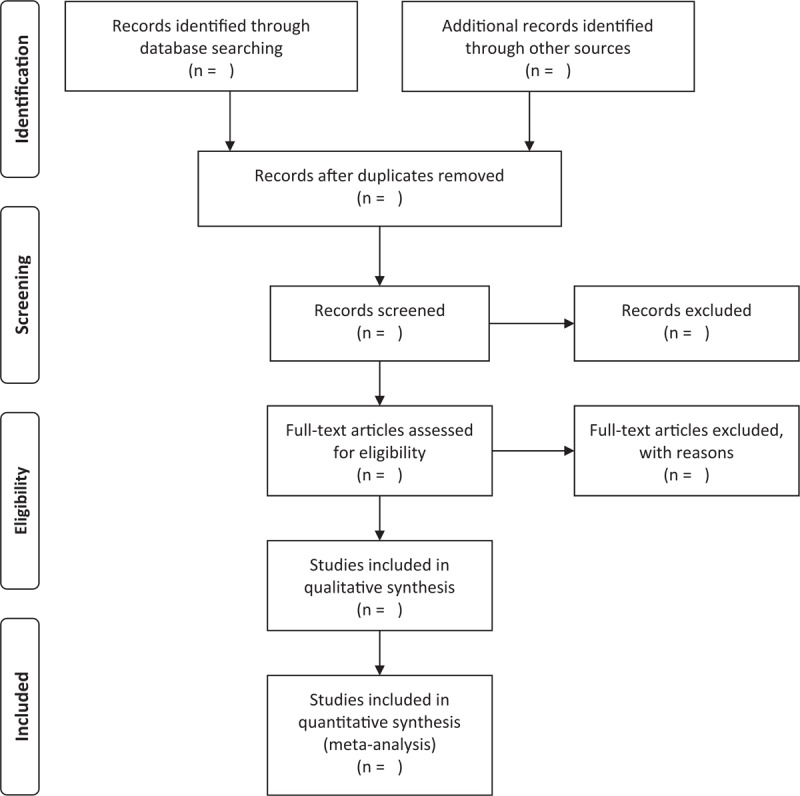
Preferred reporting items for systematic review and meta-analysis (PRISMA) flow chart.

The articles will be imported to the Endnote (version 9.0, Thomas Reuters, CA) and duplicate studies will be excluded. Later, 2 authors will read the title and abstracts to exclude the studies clearly not meeting the inclusion criteria independently. Then, the 2 authors will read the full text of literature to determine whether to include them. Finally, results will be double checked for accuracy. Whenever there is a disagreement, it will be solved by discussion or consulting a third author. The process of studies selection and meta-analysis is presented in an adapted preferred reporting items for systematic review and meta-analysis (PRISMA) flow diagram (Fig. 1).

#### 2.3.2 Data extraction and management

The data extraction will be carried out independently by 2 authors via a standardized data abstraction form. The extracted content mainly includes: basic information of the study (including title, author, publication year, journal, study design), basic characteristics (including sample size, age, sex, etc.), specific details of the intervention and treatment, outcome indicators and outcome measurement data, key factors of bias risk assessment (including random methods, blind methods, etc.). If there is a disagreement, it will also be solved by discussion or consulting a third author. The data will be double checked for accuracy.

#### 2.3.3 Assessment of risk of bias in included studies

The risk of bias of included literature will be assessed according to the bias risk assessment tool recommended by Cochrane Reviewer's Handbook V.5.3, including 7 domains: random assignment method, allocation concealment, blinding of patients, blinding of evaluator of outcome, incomplete outcome data addressed (i.e., whether to describe the loss of follow-up, the number of exits, whether an intentional analysis was conducted), selective reporting, other bias. The quality of included literature will be judged as “low risk,” “high risk,” and “unclear” from the above 7 aspects. If the opinions are inconsistent, an agreement will be reached through discussion or consulting a third author.

#### 2.3.4 Measures of treatment effect

The dichotomous outcomes will be expressed by relative risk (RR) or odds ratio (OR) with 95% confidence interval (CI). For continuous outcomes, mean difference (MD) with 95% CI will be presented if the outcome measures of all studies are based on the same unit of measurement, otherwise, standardized mean difference (SMD) with 95% CI will be presented for analysis.

#### 2.3.5 Dealing with missing data

If the necessary information of the included study is unknown or lacking, we will contact the author via email or telephone to obtain it. If it is not available, the existing data will be processed by using data synthesis, and the impact of the missing data will be discussed.

#### 2.3.6 Assessment of heterogeneity

The heterogeneity will be assessed by chi-squared test and *I*^2^ statistic. If *P* > .1, *I*^2^ <50%, there is no or low statistical heterogeneity. If *P* < .1, *I*^2^ ≥50%, statistical heterogeneity will be considered significant and further subgroup analysis or sensitivity analysis will be performed to find the source of heterogeneity.

#### 2.3.7 Assessment of reporting bias

If there are >10 studies included, a funnel plot will be used to analyze whether there is a publication bias.

#### 2.3.8 Data synthesis

RevMan5.3 software (Version 5.3, Copenhagen: The Nordic Cochrane Center, The Cochrane Collaboration, 2014) will be used to compute the data synthesis.

If the heterogeneity is low or no statistical heterogeneity (*P* > 0.1, *I*^2^ <50%), a fixed effect model will be used for data synthesis. If there is a high statistical heterogeneity (*P* < 0.1, *I*^2^ ≥50%), further analysis will be conducted to find whether there is statistical or clinical heterogeneity, a random effects model will be applied after eliminating the impact of significant clinical heterogeneity. If there is a significant clinical heterogeneity, heterogeneity and inability to judge the source of heterogeneity, descriptive analysis will be used.

#### 2.3.9 Subgroup analysis

Subgroup analysis will be used to find the source of heterogeneity while there is a significant heterogeneity, according to the evaluation criteria, age, sex, severity of the disease, dosage, and course of treatment, etc.

#### 2.3.10 Sensitivity analysis

If there is significant heterogeneity due to the different methodological quality of the included studies, the low-quality study will be excluded to determine the stability of the results.

#### 2.3.11 Grading the quality of evidence

We will evaluate the quality of evidence by using the Grading of Recommendations Assessment, Development and Evaluation (GRADE) software (Version 3.6, The GRADE Working Group, 2010). The quality of evidence was divided into 4 levels: high, medium, low, and extremely low.

## 3 Discussion

Sepsis is the most common critical illness in the ICU, with high morbidity and high mortality. Modern medicine has made some progress in the methods and means of treating sepsis, however, the incidence and mortality of sepsis are still high, which is a worldwide problem.^[22,23]^ Some clinical trials have shown that QWBDD may inhibit inflammatory reaction and regulating immunity^[18–21]^ in patients with sepsis, however, the results were inconsistent, and so far no systematic review and meta-analysis of QWBDD in treatment of sepsis has been found, which has certain limitations on clinical guidance. Therefore, we intend to conduct a systematic review of QWBDD for sepsis in order to provide high-quality evidence of effects and safety of QWBDD for sepsis, and provide reference for scientific researchers and health policy makers. However, there may be some limitations in our reviews. Firstly, only studies published in Chinese and English will be included, which may increase the bias. Secondly, there may be a heterogeneity risk due to different nationalities, doses of herbs, age of the patient, and the small sample of the included study.

## Author contributions

**Conceptualization:** Jingya Zhong, Hairong Cai.

**Data curation:** Yonglian Huang.

**Funding acquisition**: Ping Wang.

**Investigation:** Dongjie Chen.

**Methodology:** Yaxiu Huang.

**Project administration**: Xiaohong Peng.

**Software:** Shaoping Li.

**Supervision:** Xiaohong Peng.

**Validation:** Qifeng Cao.

**Writing – original draft:** ping wang, Hefei Huang.

## Supplementary Material

Supplemental Digital Content
