# Supplementary material for: Qinwen Baidu decoction for sepsis: A protocol for a systematic review and meta-analysis
Source: Medicine (Baltimore). 2019 Mar 1;98(9):e14761. doi: 10.1097/MD.0000000000014761 (PMC6831360; doi:10.1097/MD.0000000000014761)
Supplement: Supplemental Digital Content [file medi-98-e14761-s001.doc]

**Appendix A.**

***Search strategy used in PubMed database***

#1 sepsis OR Severe Sepsis OR Pyemia OR Pyohemia OR Pyaemia OR Septicemia OR Blood Poisoning

#2 qingwen baidu decoction OR qing wen bai du decoction OR qingwen baidu tang OR qingwen baidu yin

#3 Randomized controlled trial OR clinical study OR Clin-ical Trial OR Controlled study OR Controlled Trial OR Random*Control* study OR random* Control* Trial

#1 AND #2 AND #3
